# Functional properties of equine adipose-derived mesenchymal stromal cells cultured with equine platelet lysate

**DOI:** 10.3389/fvets.2022.890302

**Published:** 2022-08-09

**Authors:** Alina Hagen, Sabine Niebert, Vivian-Pascal Brandt, Heidrun Holland, Michaela Melzer, Axel Wehrend, Janina Burk

**Affiliations:** ^1^Equine Clinic (Surgery, Orthopedics), Justus-Liebig-University Giessen, Giessen, Germany; ^2^Saxon Incubator for Clinical Translation (SIKT), University of Leipzig, Leipzig, Germany; ^3^Clinic for Obstetrics, Gynecology and Andrology of Large and Small Animals, Justus-Liebig-University Giessen, Giessen, Germany

**Keywords:** mesenchymal stromal cells (MSC), platelet lysate, equine, cell fitness, functional properties, co-cultivation

## Abstract

Successful translation of multipotent mesenchymal stromal cell (MSC)-based therapies into clinical reality relies on adequate cell production procedures. These should be available not only for human MSC, but also for MSC from animal species relevant to preclinical research and veterinary medicine. The cell culture medium supplementation is one of the critical aspects in MSC production. Therefore, we previously established a scalable protocol for the production of buffy-coat based equine platelet lysate (ePL). This ePL proved to be a suitable alternative to fetal bovine serum (FBS) for equine adipose-derived (AD-) MSC culture so far, as it supported AD-MSC proliferation and basic characteristics. The aim of the current study was to further analyze the functional properties of equine AD-MSC cultured with the same ePL, focusing on cell fitness, genetic stability and pro-angiogenic potency. All experiments were performed with AD-MSC from *n* = 5 horses, which were cultured either in medium supplemented with 10% FBS, 10% ePL or 2.5% ePL. AD-MSC cultured with 2.5% ePL, which previously showed decreased proliferation potential, displayed higher apoptosis but lower senescence levels as compared to 10% ePL medium (*p* < 0.05). Non-clonal chromosomal aberrations occurred in 8% of equine AD-MSC cultivated with FBS and only in 4.8% of equine AD-MSC cultivated with 10% ePL. Clonal aberrations in the AD-MSC were neither observed in FBS nor in 10% ePL medium. Analysis of AD-MSC and endothelial cells in an indirect co-culture revealed that the ePL supported the pro-angiogenic effects of AD-MSC. In the 10% ePL group, more vascular endothelial growth factor (VEGF-A) was released and highest VEGF-A concentrations were reached in the presence of ePL and co-cultured cells (*p* < 0.05). Correspondingly, AD-MSC expressed the VEGF receptor-2 at higher levels in the presence of ePL (*p* < 0.05). Finally, AD-MSC and 10% ePL together promoted the growth of endothelial cells and induced the formation of vessel-like structures in two of the samples. These data further substantiate that buffy-coat-based ePL is a valuable supplement for equine AD-MSC culture media. The ePL does not only support stable equine AD-MSC characteristics as demonstrated before, but it also enhances their functional properties.

## Introduction

Mesenchymal stromal cells (MSC) offer regenerative properties, but their potency depends on several environmental factors, including cell culture conditions. *In vitro* cultivation of MSC is inevitable to obtain a sufficiently large number of MSC for therapeutic applications, as MSC are only present in small numbers in their tissue of origin (1, 2). During this *in vitro* cultivation, the culture conditions strongly influence the cell quality and efficacy of therapy (3). The culture medium, which is required to provide the MSC with nutrients and growth factors, is one of the most important elements of the culture conditions. The current gold standard supplement to basal medium for *in vitro* cultivation of many cell types, including MSC, is fetal bovine serum (FBS) (4–7). However, the use of FBS is not without problems and a reduction or replacement of FBS was recommended by the European Medicines Agency (EMA) (8) and the International Society for Cell and Gene Therapy (ISCT) (3). As platelet lysate is a promising cell culture supplement and it already replaces FBS in human medicine in 77% of good manufacturing practice protocols (9), equine platelet lysate (ePL) could replace FBS in equine MSC culture in an analogous way. Several studies have already shown that ePL is a suitable alternative for FBS in equine MSC culture and that basic MSC characteristics are not altered with ePL (10–14). Nevertheless, conflicting results have also been reported, showing a dose-dependent proliferation of equine MSC with ePL in two studies (15, 16), with MSC proliferation decreasing at ePL concentrations higher than 30% (15). In another study, in which platelet-derived growth factors were not released by freeze-thaw cycles but by CaCl_2_ activation, an adversely altered morphology of equine BM-MSC was described (17). The studies on ePL published so far by several research groups have used neither uniform protocols for the preparation of the platelet concentrates nor for the activation or release of the platelet-derived factors, which limits the comparability between the different studies. Therefore, we have previously reported the first scalable buffy-coat-based protocol for ePL production, and we have shown that this ePL supports equine AD-MSC expansion and differentiation similar to FBS when used in the same concentration (10%) (13, 14). Yet, as MSC are capable to adapt their functions to their environment (18, 19), prior to its introduction into the clinically used protocol, it remains crucial to further investigate the effects of ePL on MSC fitness, safety and functional characteristics.

Alterations in cell fitness can be triggered by stress factors in the cell culture conditions. The same stressors can influence the cell cycle of MSC in different ways, which entail a mitotically inactive, senescent state or the programmed cell death, apoptosis (20). In contrast to this, irreversible cell damage results in necrosis, a passive cell death that is accompanied by an inflammatory reaction (21). During MSC expansion culture, stressors should be kept at a minimum, resulting in low levels of cell death or senescence.

Regarding the safety of a cell-based product, its genomic integrity is of major relevance (22). The genetic stability of human MSC after cultivation with human PL (hPL) has already been investigated several times (23–25). In this context, a comparable or even higher genetic stability was already shown with hPL compared to FBS (23, 24). However, the genetic stability of equine MSC has so far only been studied with FBS (22, 26–28). For this reason, in the present study, we analyzed the genetic stability of equine AD-MSC cultivated with either FBS or ePL.

The best-known functional properties of MSC rely on the modulation and support of other cell types by paracrine signaling and cell-to-cell contacts. Neovascularization represents a critical issue in wound healing and tissue regeneration, because anabolic processes and the survival of (cell) transplants strongly rely on sufficient oxygen and nutrient supply. Studies investigating the effect of MSC in wound healing have not yielded consistent results in clinical trials (29, 30). Nevertheless, it was shown that human MSC have a positive effect on the treatment of ischemic tissue by releasing angiogenic factors (29). In addition, others demonstrated that platelets or platelet-containing preparations can be used to promote the regenerative potential of human AD-MSC (30) and several studies have reported an improved therapeutic efficacy of MSC from different sources induced by platelet rich plasma (31–33). After numerous promising studies have reported the preservation of the basic characteristics of human BM- and AD-MSC with hPL (34–37), and functional studies using hPL (38, 39) have also been carried out in human medicine, the time has come to perform functional assays for ePL as well.

In the present study, we aimed to test whether the cell fitness and angiogenic potency of the equine AD-MSC is synergistically supported by ePL, comparing MSC cultivated with FBS or ePL in terms of their apoptotic, necrotic and senescent state, genetic stability as well as their angiogenic potency.

## Materials and methods

### AD-MSC culture, cell fitness analysis and karyotyping

#### AD-MSC culture with FBS and PL media supplements

Adipose-derived (AD-) MSC were obtained from seven horses aged 2 to 8 years (median: 5 years; IQR: 2), either after euthanasia due to unrelated reasons or within the framework of a previous study approved by the respective local authority (Landesdirektion Leipzig, TV34/13). AD-MSC isolation was performed by collagenase digestion in accordance to the protocol described in (40). The AD-MSC were expanded in FBS-supplemented culture medium and then cryopreserved in passage (P) 1, in cryomedium consisting of Dulbecco's modified Eagle's medium (DMEM, 1 g/L glucose; Gibco®, ThermoFisher Scientific, Darmstadt, Germany) with 40% FBS and 10% dimethyl sulfoxide (DMSO, Sigma Aldrich GmbH, München, Germany) using a freezing container (Mr Frosty, Nalgene, ThermoFisher Scientific, Darmstadt, Germany), and stored in liquid nitrogen.

For the current experiments, the cells were thawed and seeded in DMEM supplemented with 10% FBS (Lot: 2078409, Gibco™, ThermoFisher Scientific), 10% ePL or 2.5% ePL. To all media, 1% penicillin-streptomycin and 0.1% gentamycin were added. To ePL media, 1 U/ml heparin-natrium (B. Braun, Melsungen, Germany) was additionally added. The ePL used in this study had been produced from whole blood obtained from n = 19 horses (approved by the regional council Giessen, A14/2019) using a buffy-coat based protocol as previously described (13). This protocol included production of a platelet concentrate with 4.2-fold increased platelet concentrations and 0.4-fold decreased white blood cell concentrations, and freeze/thaw cycles to lyse the platelets before the lysates from all donors were pooled.

AD-MSC were cultivated in the respective culture media under standard culture conditions (humidified atmosphere, 37 °C, 5% CO_2_) for at least one passage prior to their use in the experiments to allow for adaptation to the culture media. All experiments were carried out using AD-MSC from n = 5 donors as biological replicates, whereby the same donors were used for the AD-MSC cell fitness analyses and the karyotyping, or the co-culture experiment, respectively.

#### Apoptosis and necrosis assay

An apoptosis and necrosis live-cell assay (catalog#: JA1011, RealTime-Glo™ Annexin V Apoptosis and Necrosis Assay, Promega, Mannheim, Germany) was performed in P3 and P5 to analyze cell death. This involved measuring the apoptosis marker phosphatidylserine on the outer cell membrane and determining the level of necrosis using a fluorescent and cell-impermeable DNA dye. For this purpose, the AD-MSC were seeded at 5,000 cells/cm^2^ in a 96-well plate and the assay was performed according to the manufacturer's instructions. On day 5, the extent of apoptosis was determined by luminescence measurement and the intensity of necrosis by fluorescence measurement, using an Infinite M PLEX plate reader (Tecan Ltd., Maennedorf, Switzerland).

#### Senescence assay

To evaluate the aging process of the AD-MSC, a cellular senescence activity assay [catalog#: ENZ-KIT129-0120, Enzo Life Sciences (ELS) AG, Lausen, Switzerland] was performed according to the manufacturer's instructions in P3 and P5. For this purpose, the AD-MSC were seeded at 3,000 cells/cm^2^ in a 12-well plate and incubated for 5 days, with a medium change on day 3. On day 5, the medium was removed and after a washing step with phosphate-buffered saline (PBS), AD-MSC were lysed on ice with a lysis buffer containing 0.5% phenylmethylsulfonyl fluoride (PMSF) and a cell scraper. The lysate was centrifuged at 14,000 x g for 10 min at 4 °C and the supernatant was frozen at −80 °C. When all samples had been collected, they were thawed and SA-β-galactosidase (SA-β-gal) activity was measured using the fluorescent substrate in the Infinite M PLEX plate reader.

#### Karyotype analyses

The equine karyotype analysis was conducted according to a previously described protocol for equine stem cells (41). Chromosome preparation was performed on P5 AD-MSC monolayer cultures in 10% FBS or 10% ePL medium, using standard cytogenetic techniques (colcemid treatment, hypotonic treatment and methanol/acetic acid fixation). GTG analyses were carried out according to (42, 43) for cytogenetic analyses on metaphase cells. In total, 125 metaphase cells per group (25 metaphase cells per sample) were analyzed to exclude clonal occurrence of chromosomal aberrations.

### Endothelial cell culture and angiogenesis assays

#### Collection of umbilical cord arteries and endothelial cell isolation

Umbilical cords from 4 foals were collected immediately after birth and 10 cm length of the umbilical cord was placed in a jar with PBS and 5% penicillin-streptomycin for transport. In the laboratory, the umbilical cord was first washed in 70% ethanol and then again in PBS. Next, the vein and Wharton's jelly tissue were removed and the umbilical arteries were carefully dissected. One artery was used to recover endothelial cells for co-culture experiments as described below, and the other artery was subjected to an arterial ring assay as described in Supplementary material 1. In order to obtain the endothelial cells, the respective umbilical artery was filled with collagenase I [Gibco™, ThermoFisher Scientific; 0.8 mg/ml in Hank's Balanced Salt Solution (HyClone Laboratories, Logan, Utah, USA)] and the ends were closed with clamps. Then the umbilical artery was incubated for 30 min at 37 °C and massaged twice during this time. After the incubation period, the content of the artery was transferred to a centrifuge tube and the artery was massaged again while washed with PBS, which was also transferred to the same centrifuge tube thereafter. The isolated cells were pelleted (390 x g, 5 min), washed with PBS and seeded in tissue-culture-coated flasks in FBS-supplemented medium until cryopreservation in liquid nitrogen.

#### Co-culture of AD-MSC and endothelial cells

For the co-culture, a 12-well transwell system was used and the wells representing the bottom compartment were filled with 600 μl of either rat collagen I matrix (Cultrex, Minneapolis, MN, USA) or ePL matrix. The rat collagen I matrix was prepared according to the manufacturer's instructions. The ePL matrix consisted of DMEM, 10% ePL and 1% penicillin-streptomycin, and coagulation of the matrix was allowed in the absence of heparin. The matrices were prepared 24 h before seeding the cells.

Next, P3 AD-MSC from five horses were co-cultured with P3 endothelial cells from one foal. For this purpose, 2.5 x 10^4^ endothelial cells per well were seeded on the matrices and 1.0 x 10^4^ AD-MSC were seeded per insert (0.4 μm PET insert; Falcon® Cell Culture Inserts, Corning, NY, USA). Wells with collagen matrix were filled with 10% FBS-supplemented medium and wells with ePL matrix were filled with 10% ePL-supplemented medium, and cells were incubated for three days. Controls using AD-MSC from each donor or the endothelial cells only were prepared correspondingly.

#### Vascular endothelial growth factor in ePL and cell culture supernatants

To analyze the level of vascular endothelial growth factor A (VEGF-A) in the cell culture supernatants from the co-cultivation of the endothelial cells and AD-MSC, an equine VEGF-A ELISA (catalog#: ELE-VEGFA, Ray Biotech, Norcross, GA, USA) was performed according to the manufacturer's instructions. For this purpose, the cell culture supernatants were obtained on the third day of co-cultivation and frozen at −80 °C until the assay was performed. The ELISA was read on the Infinite M PLEX plate reader. Data from the cell culture supernatants were normalized by subtracting the results from the respective cell culture media before further comparison.

#### Fluorescence staining and microscopy

For morphological evaluation, endothelial cells were stained with lectin (Lectin from Bandeiraea simplicifolia, FITC conjugated, Sigma Aldrich) and AD-MSC were stained with phalloidin (Dylight^TM^ 554 Phalloidin, Cell Signaling Technology, Danvers, MA, USA); DAPI (Carl Roth GmbH, Karlsruhe, Germany) was used for counterstaining of nuclei. The cells were fixed with 4% paraformaldehyde (Carl Roth GmbH) for 30 min. Then the endothelial cells were permeabilized with PBS (containing Ca^2+^ and Mg^2+^) + 0.25% Triton X-100 (Carl Roth GmbH) for 30 min at room temperature. Next, 5% bovine serum albumin (Carl Roth GmbH), dissolved in distilled water, was added as a blocking solution for 30 min at 37 °C. After removing the blocking solution, the lectin (0.1 mg/ml, diluted in 0.9% saline solution) was added and incubated overnight at 4 °C. The AD-MSC were stained with phalloidin (1:200) for 30 min. Cells were washed with PBS + 0.1% Triton X-100 three times for 15 min, then DAPI (1:1000) was added for 30 min for nuclear staining. Directly after staining of the endothelial cells and AD-MSC, three images were obtained from each replicate at standardized settings using a Leica DMI6000 B microscope (Leica Microsystems, Wetzlar, Germany). Photomicrographs were analyzed using Fiji ImageJ software to determine endothelial cell counts.

#### Quantitative RT-polymerase chain reaction (qRT-PCR)

The relative gene expression of angiogenesis-related factors (Table 1) in AD-MSC was analyzed by qRT-PCR. Glyceraldehyde-3-phosphate dehydrogenase (GAPDH) and hypoxanthine phosphoribosyl-transferase 1 (HPRT1) were used as reference genes. Total RNA of the AD-MSC was isolated using the RNeasy Mini Kit (Qiagen, Hilden, Germany) with an on-column DNAse treatment, according to the manufacturer's instructions. The obtained RNA was converted to cDNA using the Reverse Transcriptase RevertAid H Minus First Strand cDNA Synthesis Kit (ThermoFisher Scientific GmbH). QRT-PCR was performed using SYBR Green Mastermix (Bio-Rad, Hercules, CA, USA) and a qTowerG (Analytik Jena GmbH, Jena, Germany). In each qPCR reaction, 1 μl (corresponding to 30 ng RNA) was used as template. The primers (Table 1) were designed using PrimerBlast (NCBI) and synthesized at IDT (Integrated DNA Technologies, Leuven, Belgium). The performance of the PCR reaction was determined by a melting curve and a dilution series of pooled experimental cDNA. The relative quantification of gene expression changes was performed based on the Pfaffl formula (44), using the respective geomean CT values of all FBS control samples for normalization within the formula.

**Table 1:** Equine primers for real-time PCR.

**Gene**	**Primer sequence**	**Accession no**.	**PCR product (bp)**
**eGAPDH**	For: CGATGGTGAAGGTCGGAGTAAA	NM_001163856.1	93
	Rev: TGGCGACAATATCCACTTTGC		
**eHPRT1**	For: ATTCTTTGCTGACCTGCTGGA	XM_023634464.1	147
	Rev: AGGTCATCTCCACCAATCACTT		
**eVEGFR2**	For: TCGGAAATGACACTGGAGCC	XM_023638078.1	130
	Rev: TACACCACTCCATGCTGGTC		
**eACTA2**	For: GACCCTGTTCCAGCCATCTT	XM_001503035.6	125
	Rev: CGGAGAGGACGTTGTTAGCAT		
**eCDH5**	For: TGTGTTCACGCACCAGTTGT	XM_001495895.5	127
	Rev: GTACACGACAGAGGCATGGT		
**eHGF**	For: GAACAGTGCAGACCAATGTGC	XM_014739139.2	108
	Rev: GGGAACCAGAGGCATCGTT		
**eANGPT1**	For: CCTGATCTTACACGGTGCTGA	XM_001494946.6	122
	Rev: TCAGATTGGATGGGCCACAA		

### Statistical analysis

Statistical analysis was performed with SPSS software version 28 (IBM, Ehningen, Germany) and GraphPad Prism 9 (GraphPad Software, San Diego, CA, USA). For paired comparisons involving three different culture media, Friedman tests and subsequent *post hoc* tests with Bonferroni corrections were performed. For pairwise comparisons between passages or corresponding groups in the co-culture experiments, Wilcoxon tests were performed. Differences were considered as significant at *p* < 0.05.

## Results

### AD-MSC fitness and genetic stability

#### Apoptosis, necrosis and senescence

**Figure 1:**
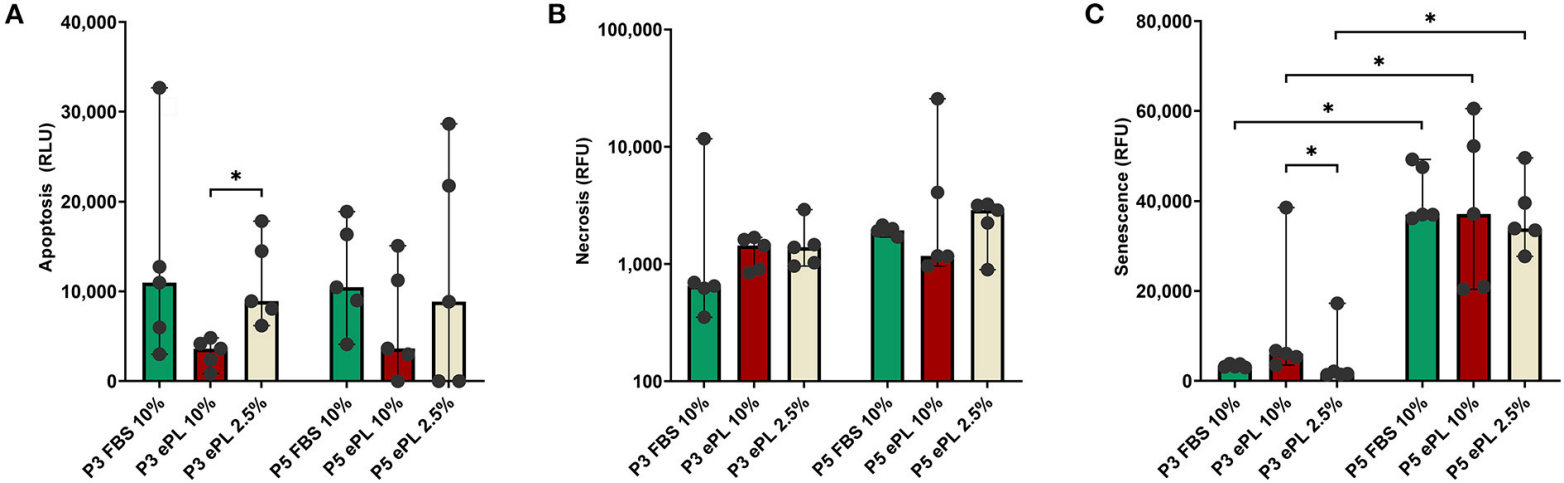
Apoptosis **(A)**, necrosis **(B)**, and senescence **(C)** levels in equine mesenchymal stromal cells (MSC) cultured with 10% fetal bovine serum (FBS), 10% equine platelet lysate (ePL) or 2.5% ePL as medium supplements, in passage (P) 3 and 5. Apoptosis was measured by a luminescence-based Annexin V assay, necrosis was measured using a cell-impermeant and pro-fluorescent DNA dye, and senescence was determined based on SA-β-galactosidase activity. The bars display the median relative fluorescence or luminescence units (RFU and RLU, respectively), error bars the 95% confidence intervals. Data were obtained with MSC from *n* = 5 donors. Friedman tests for group comparisons and subsequent *post hoc* tests with Bonferroni corrections were performed to compare the groups within P3 or P5, and Wilcoxon tests were performed to compare between P3 and P5. Asterisks mark significant differences between the indicated groups (*p* < 0.05).

The AD-MSC showed lower apoptosis levels in 10% ePL medium than in 2.5% ePL medium (*p* < 0.05 in P3). No significant differences were found regarding necrosis. The SA-β-gal activity, indicating senescence, strongly increased from P3 to P5 in all three media (*p* < 0.05). Surprisingly, AD-MSC cultivated with 2.5% ePL medium displayed the lowest SA-β-gal activity (*p* < 0.05 for 2.5% ePL vs. 10% ePL in P3) (Figure 1).

#### AD-MSC genetic stability

**Figure 2:**
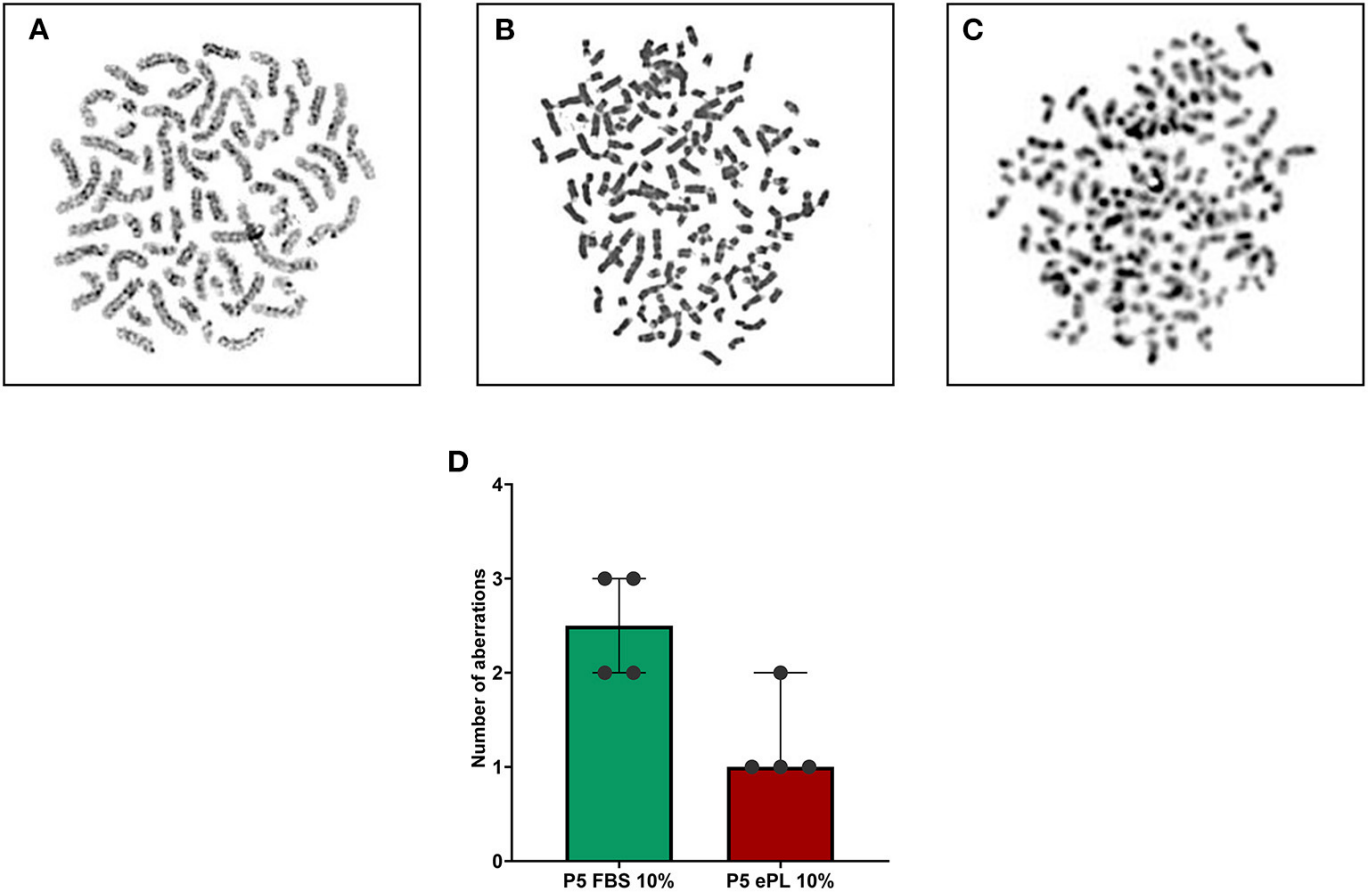
GTG karyotype analyses on metaphases from equine mesenchymal stromal cells (MSC) after cultivation in 10% fetal bovine serum (FBS) or 10% equine platelet lysate (ePL). The upper images display examples of metaphase cells with a normal karyotype **(A)**, polyploidy **(B)**, and premature centromeric division **(C)**. The bar plot **(D)** displays the number of abnormal cells found per 25 metaphase cells from each sample (*n* = 5 per group; the difference was not statistically significant).

Applying cytogenetic analyses (GTG), equine AD-MSC cultured with 10% ePL showed chromosomal aberrations in 6 out of 125 (4.8%) analyzed metaphases. Chromosomal aberrant metaphases included polyploidy (5 out of 6 aberrant metaphase cells) and premature centromeric division (PCD; 1 out of 6 aberrant metaphase cells). According to the international system for human cytogenomic nomenclature (ISCN 2020), the PCD may be used to describe premature separation of centromeres in metaphases (45). The PCD may affect one or more chromosomes in a fraction of the cells. AD-MSC cultured with 10% FBS showed chromosomal aberrations in 10 out of 125 (8%) analyzed metaphases, at which polyploidy was detected in all chromosomal aberrant metaphases. Nevertheless, the results did not suggest the presence of clonal numerical and/or structural chromosomal aberrations in the analyzed metaphases, neither in AD-MSC cultured with ePL nor in AD-MSC cultured with FBS. Figure 2 illustrates the observed aberrations and displays the numbers of aberrations found per donor.

### Pro-angiogenic effects of ePL and AD-MSC

#### Vascular endothelial growth factor in ePL and cell culture supernatants

**Figure 3:**
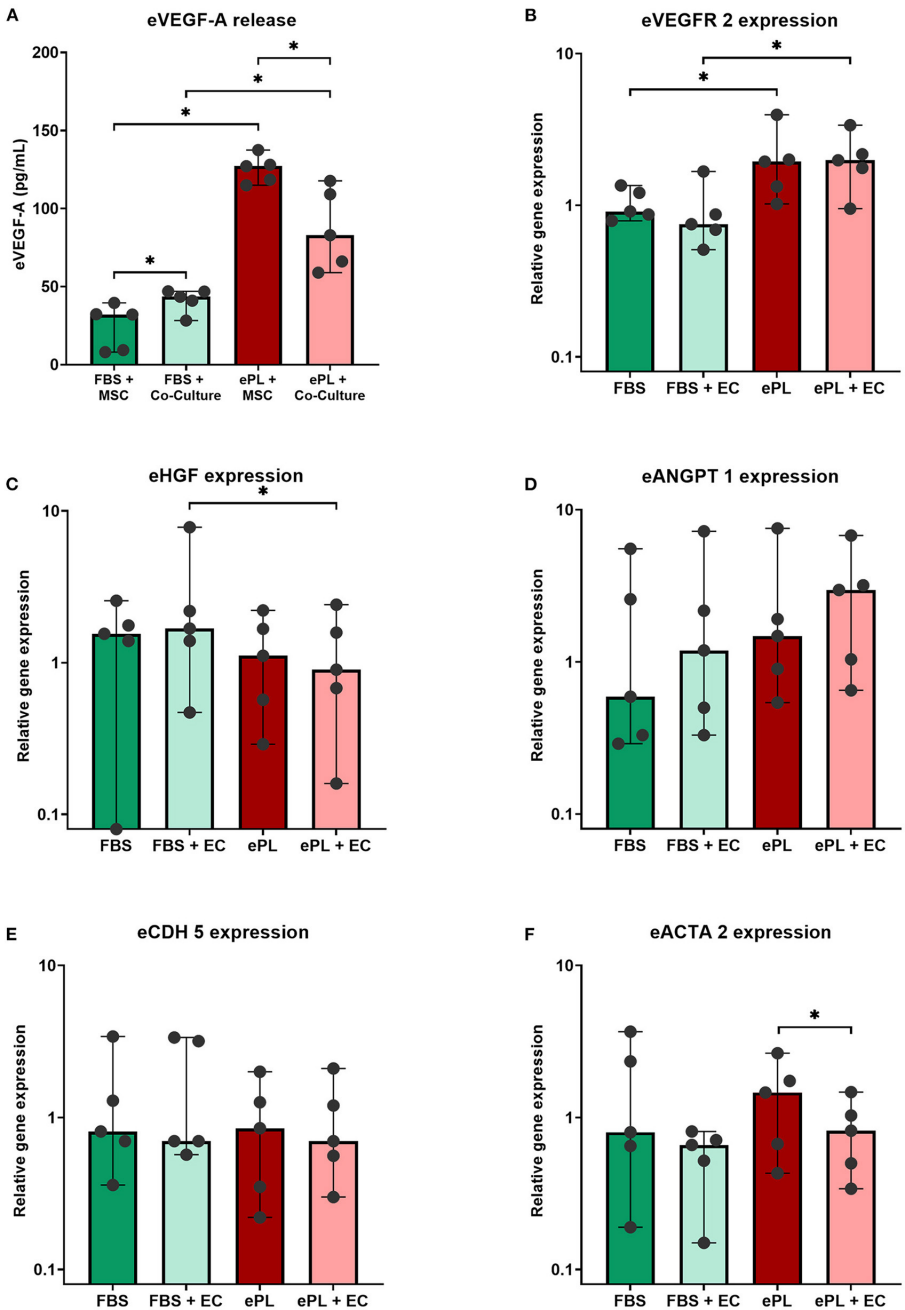
Vascular endothelial growth factor (VEGF-A) **(A)** released into the supernatants and relative expression of angiogenesis-related genes in equine mesenchymal stromal cells (MSC) **(B–F)**, after MSC were cultured alone or in co-culture with endothelial cells (EC), either with 10% fetal bovine serum (FBS) and collagen matrix or 10% equine platelet lysate (ePL) and ePL matrix. Bars display the median values and error bars the 95% confidence intervals. Wilcoxon tests were performed to compare the corresponding groups. Asterisks mark significant differences between the indicated groups (*p* < 0.05). With respect to VEGF-A release, it is of note that the data shown were normalized by subtracting the results obtained with FBS- or ePL-supplemented medium alone, thus they are indicative for VEGF-A released by the cells and from the ePL matrix. Endothelial cells from n = 1 donor were co-cultured with MSC from *n* = 5 donors.

The 10% ePL medium contained 246 pg/mL VEGF-A, but no VEGF-A was detected in 10% FBS medium. Analyzing the cell culture data normalized to the respective medium, it was evident that AD-MSC released VEGF-A in all conditions, while no VEGF-A was measured in supernatants from endothelial cells with FBS medium and collagen matrix (data not shown). The cell culture supernatants from the groups with ePL medium and matrix showed significantly higher VEGF-A concentrations than with FBS medium and collagen matrix (*p* < 0.05 for both AD-MSC alone and co-culture), which may be due to higher VEGF-A release by the AD-MSC as well as VEGF-A release from the ePL matrix. The concentration of VEGF-A was highest after the co-cultivation of AD-MSC and endothelial cells in 10% ePL medium and ePL matrix (*p* < 0.05 compared to AD-MSC alone in 10% ePL medium and matrix), and lowest after co-culture in 10% FBS medium and collagen matrix (*p* < 0.05 compared to AD-MSC alone in 10% FBS and collagen matrix) (Figure 3A).

#### Expression of angiogenesis-related genes in AD-MSC

AD-MSC showed an increased gene expression of VEGFR2 (Figure 3B), encoding for VEGF receptor-2, with 10% ePL medium and matrix, both in AD-MSC alone and in co-culture with endothelial cells (*p* < 0.05), demonstrating their response to the higher VEGF-A levels in these culture conditions.

The gene expression of CDH5, encoding for the endothelial cell junction marker VE cadherin, was not different between groups (Figure 3E). The expression of ACTA2, encoding for the pericyte-related α-smooth muscle actin (α-SMA), was highest in AD-MSC cultured alone with 10% ePL but decreased in the respective co-culture (*p* < 0.05) (Figure 3F).

No major differences between groups were observed in the expression of HGF and ANGPT1, genes encoding for the pro-angiogenic hepatocyte growth factor and angiopoietin-1 (Ang-1), respectively. The expression of HGF was lower in the ePL co-culture than in the FBS co-culture, but despite statistical significance (*p* < 0.05), this difference was relatively small (Figure 3C). ANGPT1 expression increased in the presence of ePL and AD-MSC, but this in turn was not significant (Figure 3D).

#### Effects of ePL and AD-MSC on endothelial cell growth and morphology

**Figure 4:**
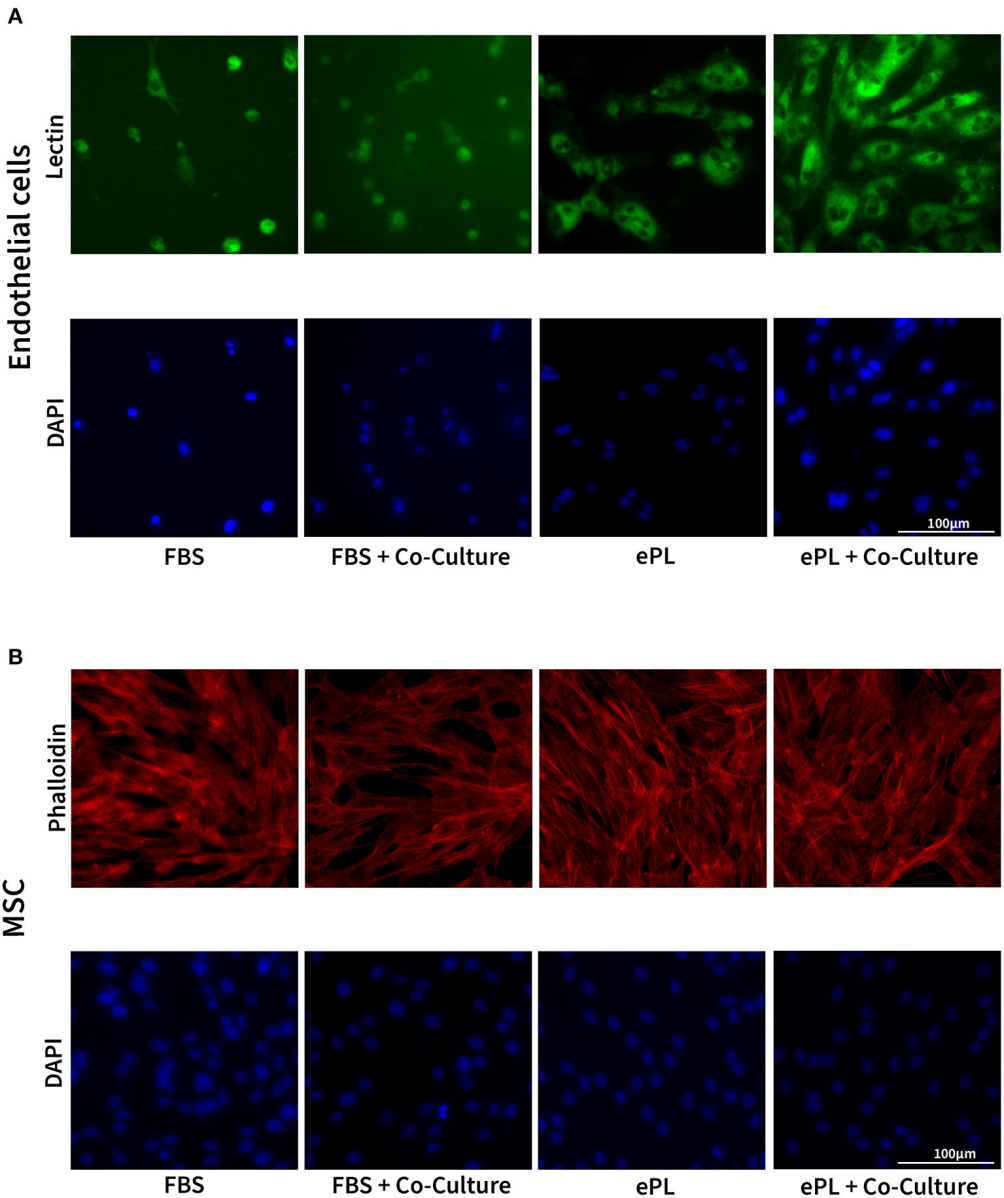
Representative images of fluorescence microscopy of equine endothelial cells **(A)** and mesenchymal stromal cells (MSC) **(B)** in indirect co-culture, cultured either with 10% fetal bovine serum (FBS) and collagen matrix or with 10% equine platelet lysate (ePL) and ePL matrix. Note the different densities of endothelial cells, while MSC morphology did not appear to be different between groups.

**Figure 5:**
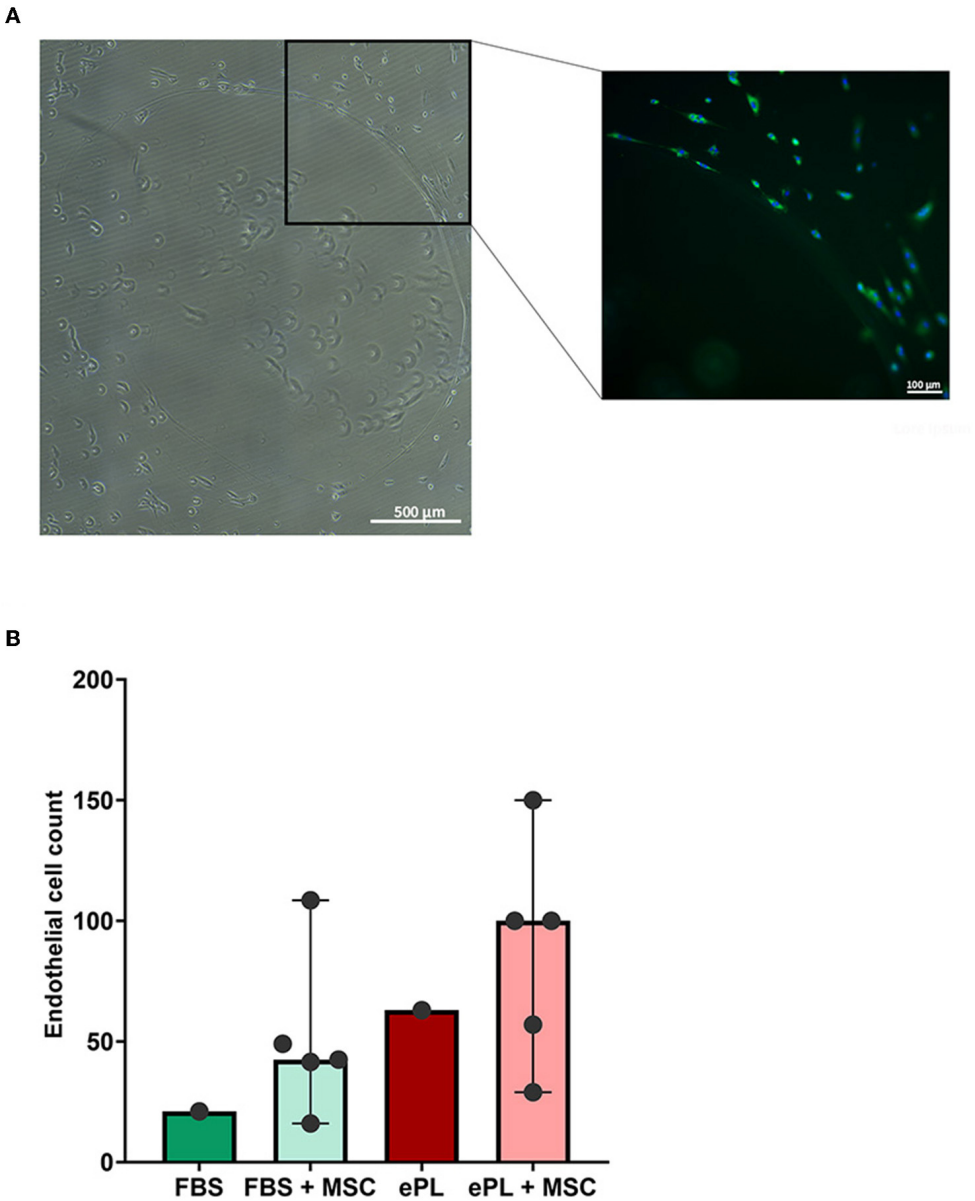
Images of equine endothelial cells displaying the formation of a vessel-like structure **(A)**, which was only observed in co-culture with mesenchymal stromal cells (MSC) and with 10% equine platelet lysate (ePL) medium and ePL matrix. To the left, a stitched phase contrast image is displayed. The image to the right presents the corresponding merged fluorescence microscopy image after lectin (green) and DAPI (blue) staining. The diagram **(B)** shows the endothelial cell counts per field of view, determined by quantitative analysis of the images obtained after co-culture with MSC. For the groups in which biological replicates in the form of co-culture with MSC from different donors were available, bars display the median values and error bars the 95% confidence intervals. Endothelial cells from *n* = 1 donor were co-cultured with MSC from *n* = 5 donors; differences were not statistically significant.

The endothelial cells appeared to be more functional with 10% ePL medium and matrix than in 10% FBS medium and collagen matrix. This was evident in the arterial ring assay, at which they exhibited more outgrowth and migration with ePL (Supplementary material 1), as well as in the co-culture assay with AD-MSC. In the latter, again more consistent endothelial cell growth was observed with ePL (Figure 4). Furthermore, the endothelial cell arrangement indicated the formation of capillaries in two of the samples co-cultured with AD-MSC and with ePL, but not in the other groups (Figure 5).

The AD-MSC showed no difference regarding cell density and cytoskeleton appearance between groups (Figure 4).

## Discussion

In this study, we demonstrated that ePL positively influences the cell fitness and genetic stability of equine AD-MSC and at the same time promotes the pro-angiogenic properties of equine AD-MSC and endothelial cells. The current work is based on a recent study (13) in which we had evaluated a protocol for the production of an ePL cell culture supplement, manufactured on the basis of a leukocyte-reduced platelet concentrate and pooled from healthy donor animals. In this previous work, we had evaluated the suitability of this ePL for equine AD-MSC culture with a focus on feasibility of MSC expansion and the minimal criteria for MSC definition, as suggested by the ISCT (46). Aiming to further ensure that the ePL does not compromise the biological and functional properties of the MSC, we now analyzed apoptosis, necrosis, senescence, genetic stability, and pro-angiogenic potency of the equine AD-MSC cultivated in FBS- or ePL-supplemented medium. This revealed that the cell culture medium supplemented with 10% ePL does not only enable equine AD-MSC expansion, but also appears to improve AD-MSC pro-angiogenic functionality.

MSC have a limited capacity for replication and after a certain number of divisions, these cells either enter a senescent state or die by programmed cell death (apoptosis) (47, 48). So far, we had observed that equine AD-MSC expansion is well feasible with culture medium supplemented with 10% ePL, but limited with 2.5% ePL, as underlined by the respective generation times and metabolic activities until P5 (13, 14). To further elucidate this issue, we here investigated equine AD-MSC senescence and apoptosis, as these stress reactions could be triggered by altered cell culture conditions (49, 50). We found that the limited proliferation observed with 2.5% ePL (13, 14) was rather due to increased apoptosis than senescence. However, we also observed a significantly increased activity of the senescence marker SA-β-gal in all media from P3 to P5. This was not unexpected, but is not fully consistent with other human studies that have analyzed senescence of human BM- and AD-MSC cultured with FBS or 5% hPL (51, 52). Surprisingly, one of these studies showed negative SA-β-gal activity up to P7 in both media (52). In the other study, SA-β-gal activity was only determined from P5 onwards, showing significantly lower activity in human BM-MSC cultured with hPL (51). Both studies concluded that senescence in human BM- and AD-MSC cultured with hPL is lower than in BM- and AD-MSC cultured with FBS, which we could not reproduce for equine AD-MSC. However, the equine AD-MSC showed lower apoptosis levels in 10% ePL medium than in 2.5% ePL medium (*p* < 0.05 in P3). Interestingly, it was shown that platelet products such as platelet lysate downregulated the pro-apoptotic factors tBid and Bim (53). At the same time, the anti-apoptotic factors BcL-xL and survivin were upregulated (53). Thus, platelet lysate seems to have a protective effect on cells against apoptosis (53, 54), which could explain our finding and is consistent with the high proliferation rates of the equine AD-MSC cultured with 10% ePL. Our results are in agreement with the findings of other equine studies comparing ePL and FBS. In this context, two studies showed similar proliferation rates with ePL and FBS (10, 11).

MSC proliferation is crucial as the cells have to be expanded *in vitro* prior to their therapeutic use, in order to obtain a sufficiently large number of cells. However, during *in vitro* expansion of cells, there is a risk of genetic transformation (50, 55). Therefore, as interest in the clinical use of equine MSC is becoming more and more widespread, the safety profile of the cultivated MSC needs monitoring. Several studies have demonstrated that human BM- and AD-MSC have a low risk of tumorigenicity (55–57), and it was shown that cytogenetic aberrations cause a reduction or arrest of cell proliferation followed by an elimination of the affected cells (22). In the study presented here, genetic stability of P5 equine AD-MSC tended to be higher after cultivation with 10% ePL than with 10% FBS. This corresponds to the findings we have already reported for canine AD-MSC and platelet lysate (14), and is consistent with studies that already demonstrated that hPL does not adversely alter the genetic stability of human BM- and AD-MSC (23, 24, 58). Nevertheless, and most importantly, neither equine AD-MSC cultured with FBS nor equine AD-MSC cultured with ePL displayed clonal aberrations, which if present would have warranted their exclusion from clinical use.

The primary mechanisms of action of MSC are based on paracrine signaling and cell-to-cell contacts. Due to the paracrine release of biologically active substances such as cytokines, chemokines or growth factors, MSC have an immunomodulating and pro-angiogenic effect and can participate in the regeneration of damaged or inflamed tissues (25, 59). Several studies have already shown that murine and human MSC of different sources migrate into ischemic tissue and improve vascular perfusion by releasing pro-angiogenic substances (29, 60–63). Vascular perfusion plays a crucial role in the regeneration of damaged tissues, as it is responsible for the oxygen and nutrient supply, as well as for the removal of waste products (64, 65). Therefore, we chose to evaluate AD-MSC functionality based on their pro-angiogenic potency.

During neovascularization, human BM-MSC support the endothelial cells by acting as perivascular cells and stabilizing the newly formed vessels (64). In this process, the human BM-MSCs were also described to differentiate into endothelial cells or smooth muscle cells by interacting with endothelial cells (64, 66, 67). Pericyte recruitment and attachment along the newly formed vessels is of tremendous importance. However, the migration of endothelial cells is independent of pericyte migration, indicating that endothelial cells and pericytes only have indirect contact *via* the basement membrane. As a consequence, communication between endothelial cells and pericytes mainly takes place *via* paracrine substances and rarely *via* direct contact (68). For this reason, we chose indirect co-culture *via* transwells in our study. This co-culture system allows an exchange of soluble substances, but no direct contact of the cells.

The paracrine factors involved in angiogenesis are HGF, VEGF, basic fibroblast growth factor (bFGF), transforming growth factor-β (TGF-β) and Ang-1 (61, 69). However, the most important growth factor for angiogenesis is VEGF-A, which is the survival signal for the endothelial cells and induces the formation of new vessels (60, 61, 68, 70). In contrast to 10% FBS medium, 10% ePL medium already contained a significant concentration of VEGF-A. Furthermore, consistent with other studies (24, 71), we observed a significantly higher VEGF-A release in the cell culture supernatants with ePL medium than with FBS medium, adding to the VEGF-A already present in the ePL-supplemented medium. In ePL medium, the VEGF-A release was further increased in co-culture, so that the highest VEGF-A concentrations were measured in the equine AD-MSC and endothelial cell co-culture with ePL medium, suggesting a synergism of ePL and AD-MSC in providing high concentrations of VEGF-A. The effect of VEGF-A is mediated *via* the VEGF-receptor-2. In accordance with others (66, 67), our data underlined the relationship between these two, as the VEGFR2 gene expression levels followed a similar trend as the VEGF-A protein concentrations.

VEGF-A has also been shown to be responsible for the differentiation of human BM-MSC into endothelial cells (66, 67), and to stimulate the proliferation (72) and tube formation of human BM- and AD-MSC (24, 66, 67). However, the low and unchanging CDH5 (encoding for VE cadherin) expression in equine AD-MSC did not support the assumption that endothelial cell differentiation had been induced in the current experiments. Based on the close relationship of equine BM- and AD-MSC and pericytes (73), MSC differentiation into this cell type might be more likely. Alpha-SMA expression is related to the regulatory function of the pericytes and involved in the control of vasoconstriction in arterioles and capillaries *via* endothelin-1 pathways (68). Co-cultivation of endothelial progenitor cells or human umbilical vein endothelial cells increased ACTA2 (encoding for α-SMA) expression in human BM-MSC in a time-dependent manner (64), indicating that BM-MSC differentiate into pericytes during co-cultivation. In the current study, we observed no increase but rather a small decrease in ACTA2 expression after co-cultivation, which could be due to the lack of the direct cell contacts previously reported as necessary for the differentiation of human BM-MSC into pericytes induced by endothelial cells (64, 74).

Finally, demonstrating equine AD-MSC functionality when cultured with ePL, their pro-angiogenic effects could be observed in the endothelial cells. Equine PL medium, together with the equine AD-MSC, appeared to provide trophic support for the endothelial cells, leading to higher cell counts in the co-culture experiments. In addition, ePL medium alone increased the outgrowth of endothelial cells in the arterial ring assay (Supplementary material 1). Last not least, although not observed in all biological replicates, only ePL and equine AD-MSC together induced tube formation in the endothelial cells. Considering these results, platelets can improve the effect of equine AD-MSC, as the pro-angiogenic potency of AD-MSC was higher with ePL medium than with FBS medium.

Despite the promising results, some limitations of the study presented here must be taken into account. For instance, the MSC were all isolated in FBS and then cryopreserved in P1. After thawing, the MSC were allowed to adapt to the ePL media for one passage, but the change of medium could still have had an impact on viability and proliferation. Another aspect worth discussing is the large variability within our data. Given that the ePL was pooled, this variability represents the differences between the AD-MSC from different donor horses, demonstrating that differences in cell quality and behavior have to be expected, irrespective of the medium used.

In summary, in this study, ePL supported cell fitness similarly as FBS. The biological properties of equine AD-MSC were positively influenced by ePL, as shown by improved pro-angiogenic effects. Thus, ePL seems to be a safe and promising cell culture supplement and a replacement of FBS by ePL in equine AD-MSC cultivation can be recommended. Moreover, based on the positive influence on the pro-angiogenic effect of equine AD-MSC, further *in vivo* studies should be conducted to establish ePL in therapeutic use alone or in combination with MSC.

## Data availability statement

The original contributions presented in the study are included in the article/Supplementary material, further inquiries can be directed to the corresponding author.

## Author contributions

AH: conception of the study and complete experimental design (together with JB), MSC culture experiments, sample and data analysis, data interpretation, and drafting of the manuscript (together with JB). SN: substantial contribution to the experimental design, MSC culture experiments, and sample and data analysis (qPCR). V-PB: sample analysis (karyotype analyses). HH: substantial contribution to the experimental design, sample and data analysis, data interpretation, and drafting of the manuscript (karyotype analyses). MM: MSC culture experiments and sample analysis (fluorescence microscopy). AW: sample acquisition (umbilical cord samples). JB: conception of the study and complete experimental design (together with AH), data interpretation and drafting of the manuscript (together with AH). All authors have critically revised the manuscript for important intellectual content and approved the publication of its content.

## Funding

This work received funding by a scholarship (AH) from the Animal Health Academy (Akademie fuer Tiergesundheit; AfT).

## Conflict of interest

The authors declare that the research was conducted in the absence of any commercial or financial relationships that could be construed as a potential conflict of interest.

## Publisher's note

All claims expressed in this article are solely those of the authors and do not necessarily represent those of their affiliated organizations, or those of the publisher, the editors and the reviewers. Any product that may be evaluated in this article, or claim that may be made by its manufacturer, is not guaranteed or endorsed by the publisher.

## Supplementary material

SUPPLEMENTARY MATERIAL 1 Arterial ring assay using equine umbilical cord arteries.
