# Supplementary material for: Functional properties of equine adipose-derived mesenchymal stromal cells cultured with equine platelet lysate
Source: Front Vet Sci. 2022 Aug 9;9:890302. doi: 10.3389/fvets.2022.890302 (PMC9395693; doi:10.3389/fvets.2022.890302)
Supplement: SUPPLEMENTARY MATERIAL 1 — Arterial ring assay using equine umbilical cord arteries. [file Data_Sheet_1.pdf]

Supplementary Material to Hagen et al.

1 Arterial Ring Assay

Method:

Fresh umbilical artery rings from n = 4 foals were cut into 3 mm thick rings. Prior to placing the arterial rings in the 24-well plate, 50 µl of either a bovine collagen matrix I (Gibco™, ThermoFisher Scientific, Darmstadt, Germany) or equine platelet lysate (ePL) matrix were added to the bottom of the wells, and further 50 µl were later added above the arterial rings. The 3 mg/ml bovine collagen I matrix was produced according to the manufacturer's instructions. The ePL matrix used in this experiment was prepared from ePL obtained by a sedimentation-based method, which involved 1 h sedimentation and then centrifugation of the obtained supernatant at 878 x g for 20 min at 22 °C to produce the platelet concentrate. This method retained more leukocytes than the buffy-coat-based method otherwise used for ePL production (Hagen et al., 2021), and we had chosen to use this type of matrix for the arterial ring assay to better mimic a wound healing environment. For ePL matrix formation, DMEM was mixed with 10% ePL, 1% penicillin-streptomycin but no heparin. The matrices were incubated for 1.5 h at 37 °C for curing. Afterwards, the culture medium was added. The arterial rings with collagen matrix were cultivated in 10% FBS-supplemented medium and those with ePL matrix were cultivated in 10% ePL-supplemented medium, using the buffy-coat-based ePL as in the other experiments. Part of the arterial rings were supplemented with 30 ng/mL equine VEGF-A (Kingfisher Biotech, Inc., St. Paul, MN, USA) and 30 ng/mL bFGF (Gibco™, ThermoFisher Scientific) once at the beginning of the cultivation period of 21 days. After cultivation, samples were stained with lectin-FITC and DAPI, and the migration of the endothelial cells from the arterial ring was examined by fluorescence microscopy and image analysis with Fiji ImageJ software.

Results:

In all groups with 10% ePL medium, more outgrowth from the umbilical arteries and superior expansion of the endothelial cells was observed. VEGF-A and bFGF supplementation tended to improve endothelial cell migration and expansion in both media, but the effect was weaker than the effect of the ePL. The highest endothelial cell densities were achieved with ePL medium + VEGF-A + bFGF (Supplementary Figure 1).

2 Supplementary Figure 1

Representative images of fluorescence microscopy of the endothelial cells migrated from the umbilical arteries, cultured in either 10% FBS **(A)** or 10% ePL medium **(B)** and supplemented with either VEGF-A or VEGF-A + bFGF. The diagram **(C)** shows the endothelial cell counts per field of view, determined by quantitative analysis of the images obtained after 21 days of cultivation. Bars display the median values and error bars the 95% confidence intervals. Arterial rings were prepared from n = 4 donors.

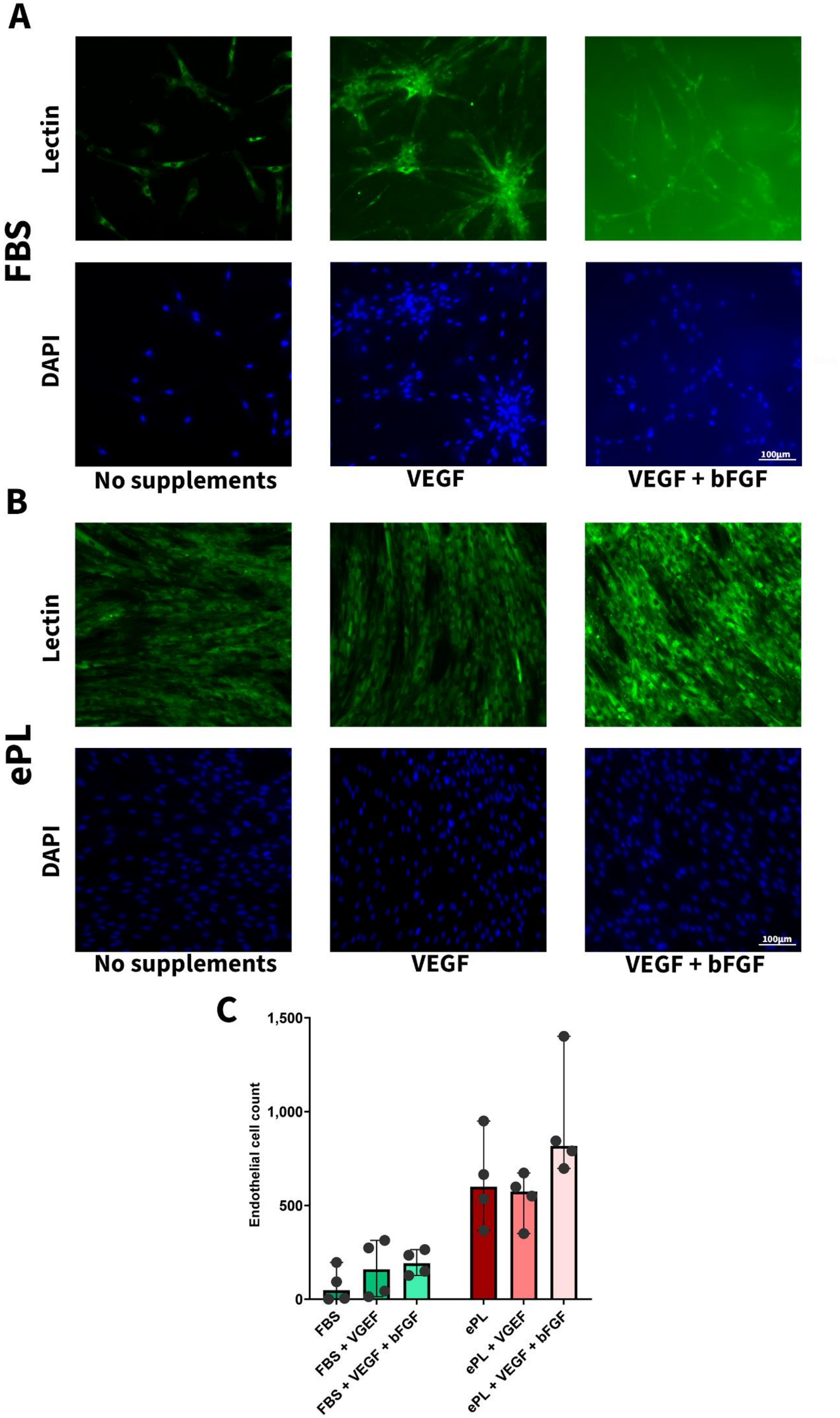
A
FBS
Lectin
DAPI
100µm
No supplements
VEGF
VEGF + bFGF
B
ePL
Lectin
DAPI
100µm
No supplements
VEGF
VEGF + bFGF
C
Endothelial cell count
1,500
1,000
500
0
FBS
FBS + VGEF
FBS + VEGF + bFGF
ePL
ePL + VGEF
ePL + VEGF + bFGF
